# Hypoxia in uterine fibroids: role in pathobiology and therapeutic opportunities

**DOI:** 10.3390/oxygen4020013

**Published:** 2024-05-28

**Authors:** Sydney L. Olson, Razeen J. Akbar, Adrianna Gorniak, Laura I. Fuhr, Mostafa A. Borahay

**Affiliations:** 1 Department of Gynecology & Obstetrics, Johns Hopkins University School of Medicine, Baltimore, MD 21205 USA; 2 Temple University, Philadelphia, PA 19122, USA

**Keywords:** hypoxia, fibroids, leiomyoma, HIF, uterine fibroids, reactive oxygen species

## Abstract

Uterine fibroids are the most common tumors in females affecting up to 70% of women world-wide, yet targeted therapeutic options are limited. Oxidative stress has recently surfaced as a key driver of fibroid pathogenesis and provides insights into hypoxia-induced cell transformation, extracellular matrix pathophysiology, hypoxic cell signaling cascades, and uterine biology. Hypoxia drives fibroid tumorigenesis through (1) promoting myometrial stem cell proliferation, (2) causing DNA damage propelling transformation of stem cells to tumor initiating cells, and (3) driving excess extracellular matrix (ECM) production. Common fibroid-associated DNA mutations include MED12 mutations, HMGA2 overexpression, and Fumarate hydratase loss of function. Evidence suggests an interaction between hypoxia signaling and these mutations. Fibroid development and growth are promoted by hypoxia-triggered cell signaling via various pathways including HIF-1, TGFβ, and Wnt/β-catenin. Fibroid-associated hypoxia persists due to antioxidant imbalance, ECM accumulation, and growth beyond adequate vascular supply. Current clinically available fibroid treatments do not take advantage of hypoxia-targeting therapies. Growing pre-clinical and clinical studies identify ROS inhibitors, anti-HIF-1 agents, Wnt/β-catenin inhibition, and TGFβ cascade inhibitors as agents that may reduce fibroid development and growth through targeting hypoxia.

## 1. Introduction

Uterine fibroids, also known as leiomyomas, are the most common tumors in females affecting up to 70% of women world-wide with increasing incidence [1,2]. Women with fibroids have significantly increased rates of infertility [3], hypertension [4], and depression [5] compared to controls. Annual costs of fibroids in the United States range from $4-9 billion for direct care, and up to an additional $17 billion for lost work-hour costs [6]. Medical treatments are limited for preventing fibroid development and growth and many people will require surgical intervention in the form of myomectomy or hysterectomy. While there is a growing body of knowledge on the pathogenesis of fibroids, many of the mechanisms remain elusive, thereby limiting the ability to develop targeted therapies. In the past decade, oxidative stress has surfaced as a key driver of fibroid pathogenesis [7]. While multiple pathways contribute to developing oxidative stress, this review will focus on hypoxia and summarize the evidence, mechanisms, and emerging hypoxia-targeting treatments in fibroids.

## 2. Hypoxia and oxidative stress

Oxidation-reduction (redox) reactions are fundamental biochemical processes that contribute to the homeostasis of living organisms. The concept of ‘oxidative stress’, initially described as an imbalance of antioxidants and oxidants, more specifically refers to a slight or severe elevation in reactive oxygen species (ROS) concentrations [8]. Specific types of ROS include any radical or nonradical derivatives of molecular oxygen (O2); predominantly superoxide (O2•-), hydrogen peroxide (H2O2), hydroxyl radical (•OH), ozone (O3), and O2 itself [9]. A disturbance in redox homeostasis, such as excessive ROS levels, can contribute to biochemical damage within cells and the development of disease [10].

Hypoxia, characterized by low oxygen levels in a living system, results in an influx of ROS by affecting complexes I, II, and III of the mitochondrial Electron Transport Chain (ETC) [11], thereby contributing to oxidative stress [12]. Under hypoxic pressure, cells activate a series of pathways to counteract the sudden decrease in oxygen including hypoxia-inducible factor (HIF), energy metabolic pathways, cell stress pathways, and autophagy [13]. In chronically hypoxic environments, many cell types deploy compensatory mechanisms to promote adaptation and survival such as angiogenesis, anerobic respiration, and glucose metabolism.

Hypoxia is implicated as a driving force in both benign and malignant conditions including atherosclerosis, diabetes mellitus, chronic obstructive pulmonary disease, Alzheimer’s disease, and numerous cancers [10]. Oxidative stress both promotes tumorigenesis via DNA damage and malignant cellular transformation and tends to create cells that can persist in hypoxic environments due to metabolic dysregulation; thus creating a self-propelling cycle of hypoxia and atypical growth [14].

## 3.0 Evidence of hypoxia in uterine fibroids

The hypothesis of hypoxia association to menstrual cycling was first proposed in the 1940s by Markee et al. who observed vasoconstriction of spiral arteries in transplanted endometrial explants following progesterone withdrawal in a Rhesus macaque model of menstruation [15]. Since then, these results have also been supported by mouse models of simulated menses that identified transient hypoxia in endometrial tissue during the menstrual phase of the menstrual cycle [16,17]. Additionally, specialized dynamic contrast-enhanced MRI protocols have been used to quantify tissue hypoxia in many organ systems, with a recent study identifying temporary endometrial hypoxia at time of menstruation in women with normal cycles [18]. In addition to spiral artery constriction, small contractions of the myometrium during menses are also hypothesized to contribute to hypoxia which may initiate local hypoxic triggered cell signaling to facilitate cessation of menses by stabilizing the endometrium [16,19].

The physiologic hypoxia of the menstrual cycle is hypothesized to be a contributor to pathologic hypoxia seen in the development and growth of fibroids. Fibroid development is multifactorial with influence from local and systemic inflammation, endocrine disrupting chemicals, altered sex hormone signaling, genetic predispositions, vitamin d deficiency, and lifestyle exposures. Many of these factors contribute to oxidative stress and result in hypoxic cellular environments that persistent beyond the menstrual cycle [20].

Based on the invasive nature of *in vivo* sampling, much of the evidence for hypoxia in fibroids depends on measurements of well establish cell signaling responses to hypoxia or ex vivo pathologic analysis. One of the few in vivo human studies used intraoperative polarographic needle electrode measurements to measure partial pressure of oxygen in leiomyoma compared to normal myometrium in women undergoing hysterectomy and found median pO2 of 1 mmHg (range 0–5 mmHg) in leiomyomas compared to 9 mmHg (range 5–20 mmHg, p < 0.0001) in surrounding myometrial tissue [21]. This direct measurement supports the many studies that have found various proteins associated with hypoxic states to be significantly altered in patients with fibroids including: HIF-1 [22–24], ET-1 [25], ALDOA, ENO1, LDHA, VEGFA, PFKFB3, and SLC2A1 [23], VEGF-A, ADM [24], COX-2 [22], catalase [26], and additional genes well summarized by Fedotova et al. [27].

## 4.0 Hypoxia and the development of fibroids

**Figure 1.**
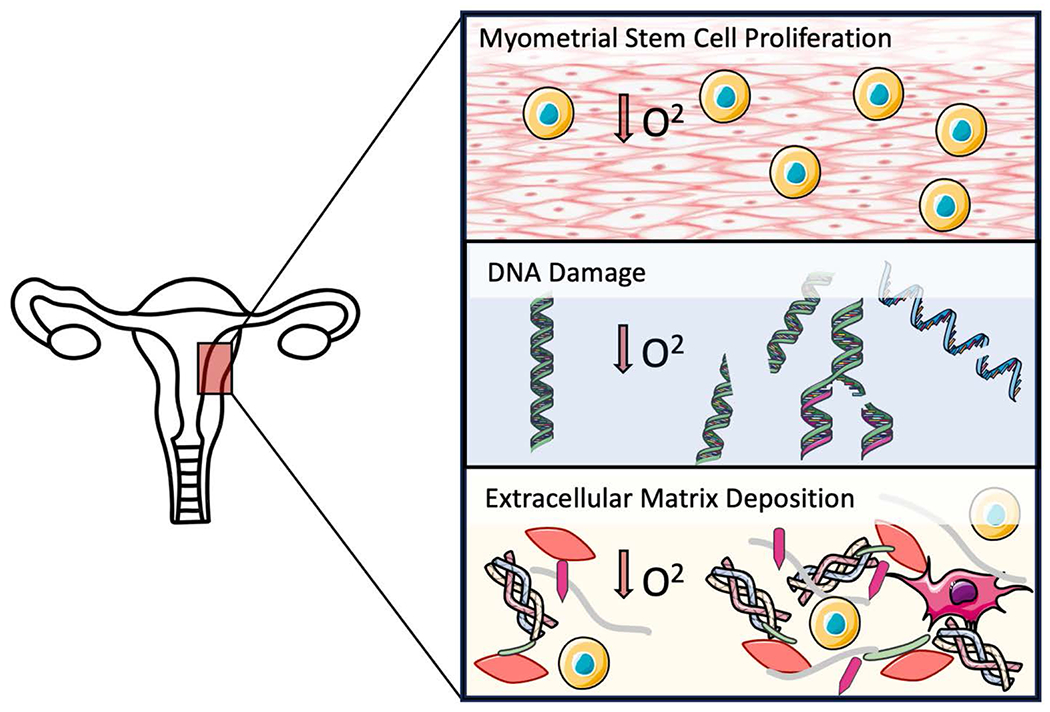
Mechanisms of hypoxia in the development of uterine fibroids. In low oxygen environments, hypoxic conditions activate myometrial stem cells (MSCs) (yellow cell) proliferation (top box). Hypoxia induces DNA damage through various mechanisms including single and double stranded DNA breaks, nitrogen base damages, mismatched bases, and base degradation (middle box). All these mechanisms of DNA damage have the ability to cause genetic alterations transforming MSCs into tumor-initiating stem cells. Extracellular matrix normally contains collagen (tricolored bundle), fibronectin (green), proteoglycans (grey), laminins (pink), between smooth muscle myometrial cells (red cell) and MSCs (yellow cell). Hypoxia induces increased ECM production through increased synthesis of ECM components as well as increased activity of enzymes that modify the ECM increasing cross links and stiffness (bottom box). Fibroblast cell types (dark pink cell) also contribute to fibrosis of the ECM under hypoxic conditions.

Hypoxia is involved in key events that contribute to the development of fibroids, as well as in signaling that promotes additional growth and persistence of cells. While the transformation and growth of fibroids exists in a continuous spectrum, the following section will focus on hypoxia contributing to inciting events in fibroid development (Figure 1) [19, 28–30].

### 4.1. Uterine fibroid stem cells

Myometrial stem cells (MSCs) have been isolated from normal myometrial tissue and are characterized by a lack of myometrial cell markers, expression of CD44(Stro-1) [31], and ability to proliferate and differentiate into mature myometrial cells, osteocytes, and adipocytes [28]. These cells contribute to hypertrophy and hyperplasia during pregnancy as well as oxytocin receptor expression [28]. Growing evidence supports MSCs as the origin cell for leiomyomas with acquisition of cellular mutations (discussed in next section) that reprogram the cells into tumor-initiating stem cells (TICs) resulting in a terminally differentiated subset of monoclonal cells that give rise to leiomyomas [32,33].

Multiple lineages of stem cells (mesenchymal, neural, hematopoietic) have been shown to preferentially reside in hypoxic niche environments [34]. One hypothesis is relative hypoxia allows stem cells to avoid oxidative damage and growth pressure thereby maintaining pluripotency. Specifically, myometrial stem cells have been shown to require hypoxic environments in vitro to proliferate [28]. This finding supports a theory that uterine environments with greater degrees or persistence of hypoxia enable greater MSC proliferation, thereby increasing the possibility of MSC transformation into a TIC. This hypothesis is further supported by the finding that women with fibroids had significantly larger populations of MSCs in their myometrium than women without fibroids [35].

### 4.2. Genetic mutations

**Figure 2.**
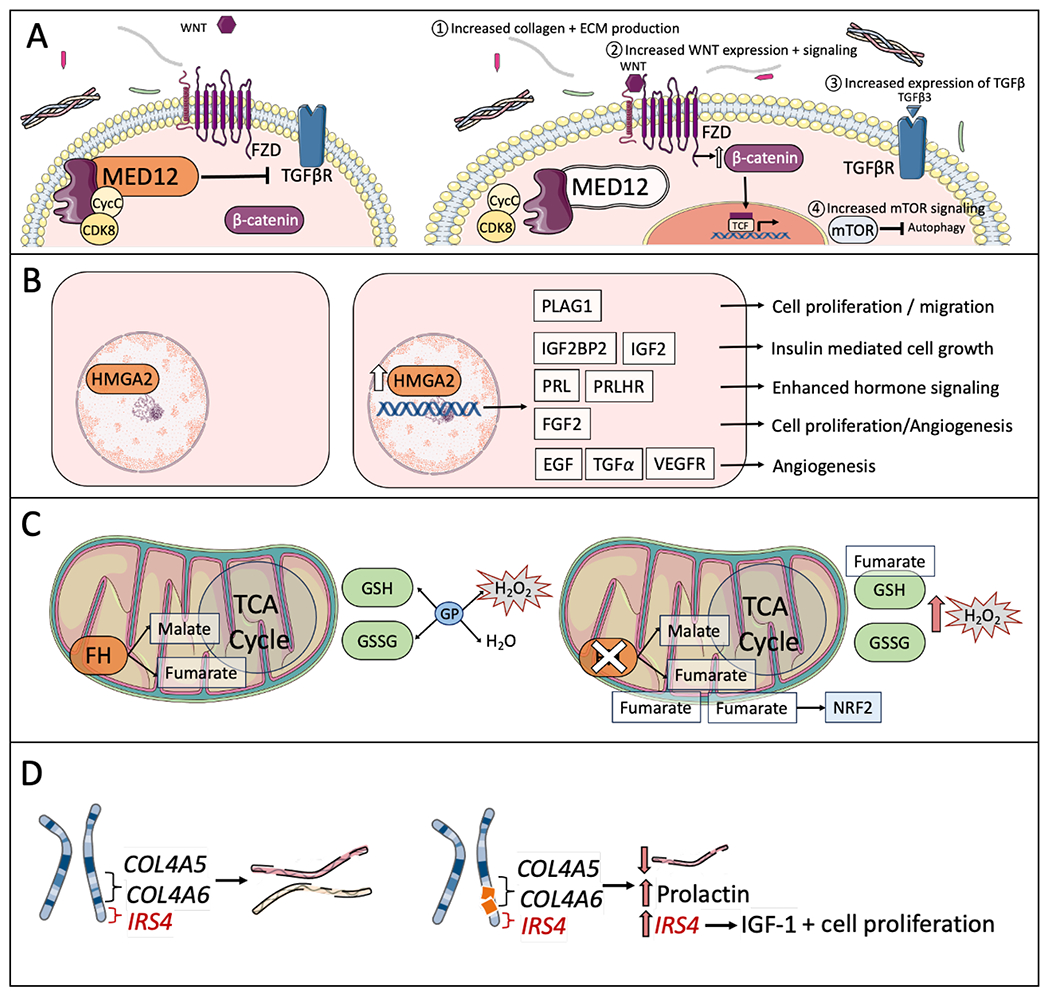
Cellular mechanisms of the common genetic mutations in leiomyomas. (A) MED12 missense mutation: Mediator of RNA polymerase II transcription, subunit 12 homolog (MED12) normally acts as one of up to 30 proteins to form the mediator complex (purple protein) that modulates RNA polymerase activity through multiple mechanisms. MED12 directly interacts with Cyclin C (CycC) and cyclin-dependent kinase 8 (CDK8) to maintain mediator activity. Wild-type MED12 is associated with TGFβR inhibition. When mutated, MED12 exhibits gain of function features in fibroids. Downstream effects include 1) increased collaged and extracellular matrix (ECM) production, 2) increased WNT expression and signaling through Frizzled family receptor (FZD) resulting in accumulation of β-catenin. β-catenin translocates to the nucleus and binds T-cell factor (TCF) and upregulates genes involved in cell growth, 3)Increased expression of pro-growth and fibrotic signal cascade Transforming growth factor β (TGFβ), 4) increased signaling through mechanistic target of rapamycin (mTOR) which inhibits autophagy in fibroids. (B) HMGA2 overexpression: high mobility group AT-hook 2 (HMGA2) binds to AT rich DNA sequences to maintain chromatin structure and regulate transcription. Overexpression of HMGA2 results in increased expression of downstream targets including proto-oncogene pleomorphic adenoma gene 1 (PLAG1), insulin-like growth factor-2 mRNA-binding proteins 2 (IGF2BP2), Insulin-like growth factor-2 (IGF2), prolactin (PRL), prolactin releasing hormone receptor (PRLHR), fibroblast growth factor 2 (FGF2), Epidermal growth factor (EGF), Transforming growth factor α (TGFα), vascular endothelial growth factor (VEGF). (C) FH loss of function: Fumarate hydratase (FH) converts fumarate to malate as one step in the tricarboxylic acid (TCA) cycle of aerobic metabolism. Deficiencies in FH lead to a buildup of fumarate which directly binds the antioxidant glutathione. Glutathione peroxidase (GP) catalyzes the reduction of hydrogen peroxide (H2O2) to water (H2O) and oxidation of Glutathione (GSH) to glutathione disulfide (GSSG); deficient quantities of GST lead to accumulation of ROS. (D) COL4A5/COL4A6 microdeletion / rearrangement: COL4A5 and COL4A6 are alpha chains of type IV collagens that contribute to structure of the fibroid ECM. When microdeletions and rearrangements in these genes occur, a downstream sequence encoding insulin-receptor substrate 4 (IRS4) is upregulated. IRS4 contributes to increased insulin-like growth factor I (IGF-1) which is known to contribute to uterine fibroid development and growth. Prolactin and COL4A5 are also significantly increased in collagen mutated fibroids compared to other mutation types and to normal myometrial cells.

Uterine fibroids, like other tumors, undergo genetic variations through DNA mutation. Mechanisms of ROS inducing DNA damage include single and double stranded DNA breaks, nitrogen base damages, mismatched bases, and base degradation [29,30,36]; these well-studied mechanisms of damage paired with increased MSC activity are hypothesized to contribute to genesis events in fibroid formation [37]. Recent studies have identified 4 recurrent driver gene mutations that are suspected to occur at the onset of tumorigenesis (Figure 2): gain of function mutations in Mediator of RNA polymerase II transcription, subunit 12 homolog (MED12), high mobility group AT-hook 2 (HMGA2) rearrangements, biallelic inactivation of fumarate hydratase (FH), and deletions of collagen, type 4, alpha 5/6 (COL4A5/COL4A6) [38].

#### 4.2.1. MED12

MED12 is the most prevalent among these studied mutations with whole-genome sequencing finding MED12 mutations in up to 70% of uterine fibroids (Figure 2A) [7,39]. The MED12 gene, located on chromosome Xq13.1, expresses missense gain of function mutations in leiomyomas which typically occur in exon 2 [38] or rarely in the intron-2-exon-2 junction [37]. These mutations play a key role in uterine fibroid tumorigenesis [40] by altering the mediator complex responsible for activating cyclin-dependent kinase 8 (CDK8) and regulating mediator-polymerase II interactions during initiation of gene transcription [37,41,42]. The exact biologic mechanisms altered by MED12 mutations are an active field of study that may involve aberrant R-loop function and altered replication fork dynamics resulting in replication stress [43,44]. Common cellular outcomes of MED12 mutation include increased ECM deposition [45] and estrogen responsiveness, decreased autophagy, and aberrant down-stream cell signaling via Wnt/β-catenin and mTOR [46] compared to normal myometrial cells [47]. Additionally, MED12 mutations are associated with greater number of individual fibroids, subserousal fibroids [48], and are more common in black women compared to white and Asian women [49]. Within patients, individual fibroids are thought to independently occur as evidenced by unique mutation burdens, with some patients harboring up to 5 different MED12 mutations across 5 different fibroids [50].

#### 4.2.2. HMGA2

HMGA2 is part of the high mobility group family of proteins comprised of nonhistone, chromatin-associated molecules that binding to AT rich DNA sequences to maintain chromatin structure and regulate transcription (Figure 2B) [51]. In leiomyomas, HMGA2 mutations are most commonly caused by the chromosomal rearrangement of 12q15, leading to an overexpression of the protein and contributing to an individual mutation frequency of around 10-20% [38]. Though previously believed to be mutually exclusive driver gene mutations, HMGA2 and MED12-mutated uterine fibroids have been frequently reported in tandem in recent studies [52]. Downstream effects of HMGA2 include upregulation of angiogenic proteins [53], and this signaling tends to result in larger solitary tumors compared to other mutations burdens [54]. The most studied downstream targets of increased HMGA2 activity are proto-oncogene pleomorphic adenoma gene 1 (PLAG1), Insulin-like growth factor-2 (IGF2) [55], fibroblast growth factor 2 (FGF2) [56], Epidermal growth factor (EGF), vascular endothelial growth factor (VEGF) [53].

#### 4.2.3. Fumarate Hydratase

Fumarate Hydratase inactivation in both alleles is seen in 1-2% of leiomyomas (Figure 2C)f. Fumarate hydratase converts fumarate to malate as one step in the tricarboxylic acid (TCA) cycle of aerobic metabolism [57]. Deficiencies in FH lead to a buildup of fumarate which has been shown to directly binds the antioxidant glutathione and thereby contribute to an increase in ROS [58]. FH deficiency occurs both in sporadic mutations within leiomyomas [59], as well as somatic mutations in hereditary leiomyomatosis and renal cell carcinoma (HLRCC) which is characterized by heterozygous germline mutations in FH [60]. The identification of FH deficient fibroids is done at time of pathologic analysis and growing research is being conducted to characterize histologic features of this unique fibroid subset [61]. FH deficient leiomyomas have been uniquely shown to activate nuclear factor erythroid 2-related factor 2 (NRF2) target genes based on the excess of fumarate. NRF2 is an anti-inflammatory transcription factor that regulates multiple pathways involved in cells response to oxidant signaling and damage [62]. NRF2 is pathologically upregulated by many cancers as a means of promoting resistance to oxidative damage and promoting growth [63]; the exact role of NRF2 in fibroids requires further investigation but likely follows this pathway.

#### 4.2.4. Collagen

Collagen (COL4A5/COL4A6) deletions occur at a frequency of less than 1% but are observed across fibroid samples [38]. Type IV collagens, such as COL4A5 and COL4A6, are major structural components of basement membranes (BMs) that separate epithelial cells from endothelial cells [64]. The COL4A5 gene, located on chromosome Xq22 and head-to-head with the COL4A6 gene, encodes the α5 chain of type IV collagen2 which forms a heterotrimer with α3, α4, and α5 chains [65]. The COL4A5 α5 chain is largely responsible for the promotion of tumor angiogenesis and cell proliferation in lung cancer through non-integrin collagen receptor DDR1-mediated ERK activation [64]. Similarly, the COL4A6 gene encodes the α6 chain of type IV collagen, providing structural support in the BMs of heart, aorta, esophagus, and bladder [66]. According to studies conducted by Mehine and colleagues, alterations in the COL4A5/COL4A6 locus arose in multiple uterine leiomyoma samples through chromothripsis, a series of complex chromosomal rearrangements associated with aggressive cancer types and tumors, such as leiomyomas [67]. These studies further indicated an association between elevated IRS4 expression and deletions in COL4A5/COL4A6 [67]. Located adjacent to COL4A5, the IRS4 gene encodes insulin-receptor substrate 4, a downstream effector of insulin-like growth factor I known for its role in uterine fibroid development [67]. COL4A5/COL4A6 mutated leiomyomas have also been shown to have significantly elevated prolactin and associated genes in genome-wide association studies (GWAS) [55].

### 4.3. Extracellular matrix

Normal myometrium is composed of parallel smooth muscle cells and interfascicular collagens that form a basketweave appearance on histology, whereas fibroids have disordered proliferation and significantly increased deposition of collagens, fibronectin, laminins, and proteoglycans creating a fibrotic stromal whorl matrix [19,68]. Fibroid growth has been described in 4 phases: (1) proliferation of myocytes, (2) proliferation of myocytes and synthesis of collagen, (3) proliferation, synthesis of collagen, and early senescence and (4) involution [19]. In phase 1 fibroids do not have a significant concentration of collage in their matrix and by phase 4 involution fibroids are > 50% collagen. In phases 3 and 4 fibroids become removed from native vascular supplies and rates of angiogenesis fail to keep up with ECM deposition creating interstitial ischemia [69]. Additionally, fibroblast cells in the ECM secrete auto- and paracrine signals that further promote growth of leiomyoma smooth muscle cells and collagen production [70]. Larger fibroids have higher relative proportions of fibroblast cells than smaller fibroids suggesting their key role in driving growth through ECM expansion [71]. Furthermore, once formed uterine fibroids are surrounded by a pseudo-capsule composed of areolar muscle fiber that separates the internal monoclonal smooth muscle cells and excess extracellular matrix from the normal myometrium and uterine circulatory system [72,73]. Fibroid myocytes then undergo inanosis- a slow cellular death progressing over days to months due to lack of nutrients-leaving behind myocyte tombstones on electron microscopy [19].

Hypoxia again acts in a self-promoting cycle of driving cell signals that result in excessive ECM deposition, and the dense ECM furthering the hypoxic environment [74,75]. ROS are known to increase activity of lysyl oxidase (LOX) [76], an enzyme that creates intra- and inter-molecular cross-links on collagen in the ECM. In fibroids, LOX expression is increased and drives excess lysine residues on collagen creating additional hydroxylysylpyridinoline (HP) and lysylpyridinoline (LP) cross-links resulting in a stiff ECM [77]. Transforming growth factor (TGF) β signaling (one of the pathways to be discussed below) is activated by hypoxia and TGFβ3 has been shown to increase mRNA expression of key ECM components including collagen 1A1, connective tissue growth factor, and fibronectin [78,79] ; as well as decrease expression of enzymes involved in matrix resorption matrix metalloproteinase 2 and 11. [79] In addition to being a physical barrier slowing oxygen diffusion, the dense ECM increases the stiffness of the myometrium and mechanotransduction transmits this biomechanical stress throughout the uterus [20]. Mechanotransduction, conversion of physical stretch signals into biologic signals, is hypothesized to contribute to rates of subfertility seen in patients with fibroids due to propagation of signals that disrupt endometrial proliferation [80].

## 5. Hypoxia-associated cell signaling in uterine fibroids

### 5.1. HIF

Production of transcription factor hypoxia-inducible factor-1 (HIF-1) is one of the most common signaling mechanisms to hypoxia seen throughout the body [81]. HIF-1 consists of two subunits, alpha and beta, located in the cells’ cytoplasm and nucleus respectively. HIF-1α activity is dependent on cellular oxygen status, while HIF-1β is constitutively expressed. In conditions with normal oxygen, HIF-1α activity is regulated by prolyl-4-hydroxylases (PHD) and factors inhibiting HIF (FIH). Both PHDs and FIHs require oxygen, thus, in hypoxic conditions, these inhibiting factors are unfunctional and the HIF-1α subunit can translocate into the cells’ nucleus and form a heterodimeric complex with the beta subunit and modulate the expression of HIF responsive genes.

HIF-1 induces more than 100 target genes which are important in hypoxic adaptive pathways, such as angiogenesis, oxygen homeostasis, and cell proliferation [23,82]. In a study performed by Miyashita-Ishiwata et al., the researchers found that under hypoxic conditions, HIF-1 was expressed in both leiomyoma and normal myometrium; however, secretion of HIF-1 target proteins, such as VEGF-A, ET-1, and ADM was found only in leiomyomas [24]. This suggests that uterine fibroids have developed an adaptive response to hypoxia. Similarly, Ishikawa et. al. found six HIF-1 target genes (ALDOA, ENO1, LDHA, VEGFA, PFKB3, and SLC2A1) that were significantly upregulated in uterine fibroids [23]. Of these target genes, vascular endothelial growth factor (VEGF-A), adrenomedullin (ADM), and endothelin 1 (ET-1) are involved in angiogenesis allowing the fibroid to divert nutrient and oxygen and continue to grow [23,24]. Aldolase A (ALDOA), lactate dehydrogenase A (LDHA), pyruvate kinase M (PKM2), and 6-phosphofructo-2-kinase/fructose-2,6-biphosphatase 3 (PFKFB3) are HIF response targets that regulate efficient anaerobic cell metabolism via glucose transportation and glycolysis [23]. Additional GWAS studies of fibroid samples have shown significant enrichment of genes related to angiogenesis, vascular tone, pro-growth, and anti-apoptosis [27].

### 5.2. Wnt/β-catenin

The Wingless-related integration site/β-catenin (Wnt/β-catenin) pathway is imperative to the growth, development, and proliferation of uterine fibroid cells [83]. Wnt, a glycoprotein secreted by cells into the ECM, activates the Frizzled family membrane receptor initiating a signal cascade that results in inhibition of β-catenin degradation, a transcriptional regulator of downstream target genes [84,85]. Ultimately, activation of wnt in fibroids leads to increased concentrations of β-catenin in the nucleus where it binds to T-cell factor (TCF) and lymphocyte enhancement factor (LEF) transcription factors, resulting in the expression of target genes involved in cell differentiation, survival, and neoplasia [86]. Overexpression of various Wnt ligands activate the Wnt/β-catenin signaling pathway and promote the formation and growth of uterine fibroids (Wnt 4, Wnt 5A) and leiomyoma stem cells (Wnt 11, Wnt 16), respectively [83].

Target effects upregulated in fibroids by signaling through Wnt/β-catenin include: estrogen, progesterone, TGFβ, PI3K/Akt/mTOR, Ras/Raf/MEK/ERK, IGF, Hippo, and Notch signaling [83]. Mutations in MED12 are hypothesized to contribute to increased Wnt4 and β-catenin protein levels contributing to dysregulated signaling through Wnt/β-catenin [46]. The excess activation of cell growth processes through Wnt may further hypoxia and the excess ROS levels that drive DNA damage, tumor formation, and progression [87].

### 5.3. TGFβ

In addition to HIF and Wnt pathways, transforming growth factor β (TGFβ) is a well characterized signaling pathway used by many cell and tumor types driving proliferation. TGFβ, present in three main isoforms (TGF-β1, TGF-β2, TGF-β3), is a polypeptide secreted into the ECM. Signaling through the TGFβ receptor results in phosphorylation of transcription factor SMAD3, which allows it to translocate to the nucleus and upregulate target genes [88].

Overexpression of TGFβ in both uterine fibroids and the myometrium contributes to growth and symptom progression; in particular, the TGFβ3 isoform remains present in uterine fibroid tissue with concentrations nearly five-times higher than that of the normal myometrium [89–91]. Hypoxia has been shown to directly induce excess TGFβ3 expression in fibroids [92]. Downstream targets of TGFβ3/SMAD3 signaling transcribed in fibroids include collagen, plasminogen activator inhibitor (PAI) 1, and connective tissue growth factor (CTGF) [93]. TGFβ3 also drove increased expression of NADPH oxidase 4 (NOX4), a producer of ROS superoxide and H2O2, further driving local hypoxia [92,94]. TGFβ also induces fibronectin and glycosaminoglycan expression [91] in leiomyoma cells contributing to the dense ECM that further drives hypoxia [95].

### 5.4. Antioxidant signaling

In addition to pro-growth signals in the presence of hypoxia, the ability of fibroid cells to combat ROS is impaired in various pathways. Two common antioxidant enzymes superoxide dismutase-3 (SOD-3) and catalase have been shown to have significantly reduced activity and mRNA in leiomyoma cells compared to normal myometrial cells [26]. SOD catalyzes the dismutation reaction of superoxide ion (O2•−) to H2O2 and catalase scavenges H2O2 catalyzing decomposition into O2 and H2O. SOD is primarily secreted as a plasma protein and acts in the extracellular space; decreased expression in fibroids likely furthers the hypoxic environment created by the dense ECM [96]. An additional repressed antioxidant pathway is impairment of manganese superoxide dismutase (MnSOD) activity via acetylation which is seen in a majority of immortalized leiomyoma cell lines with significantly reduced function compared to patient matched myometrial cells [97]. section is not mandatory but can be added to the manuscript if the discussion is unusually long or complex.

## 6. Hypoxia as a therapeutic target in uterine fibroids

Fibroids can vary in size and location and are classified by the FIGO Leiomyoma Subclassification System. Due to their diverse presentation, symptoms vary with abnormal uterine bleeding being the most common symptom. Other presenting symptoms are collectively referred to as bulk symptoms with potential impact on the genitourinary and gastrointestinal tract as well as pelvic pressure [98,99]. Currently, fibroids leading to bothersome symptoms are managed with expectant, medical, procedural, or surgical intervention with the primary goal to reduce symptom burden. While hysterectomy is the definitive treatment option for uterine fibroids and is the leading indication for hysterectomy [98], many patients opt for alternative management options to avoid surgical intervention due to patient preference, surgical candidacy, or desire for future fertility.

**Figure 3.**
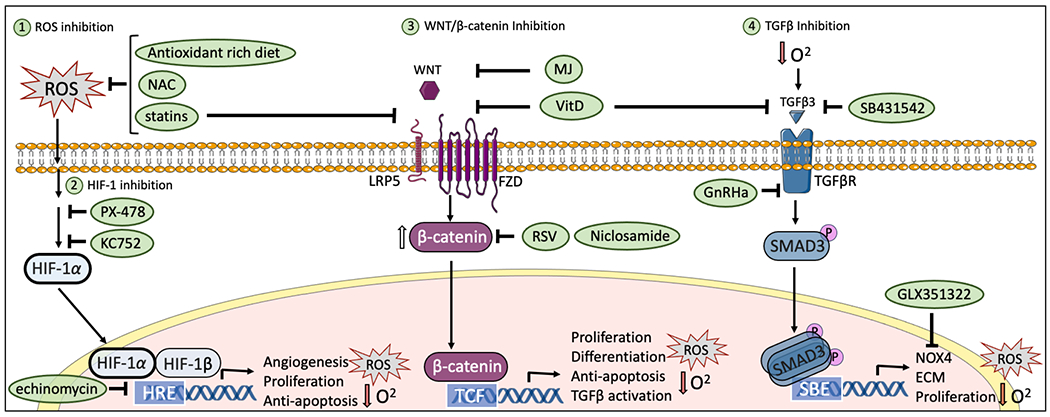
Emerging therapeutics targeting hypoxia and reactive oxygen species for treatment of uterine fibroids. (1) Targeted inhibition of ROS in leiomyoma has been shown through antioxidant rich diets, N-acetyl cysteine (NAC), and use of statins. (2) Hypoxia inducible factor 1 (HIF-1) is activated by hypoxia and ROS. PX-478 and KC752 both inhibit pathways upstream of HIF-1α resulting in lower concentrations of HIF-1α and reduced cell growth of leiomyoma cells. HIF-1α translocate to the nucleus under hypoxic conditions and binds with HIF-1β which permanently resides in the nucleus. The heterodimer locates and associates with hypoxia-responsive elements (HREs) of its target genes, resulting in transcriptional upregulation of genes involved in angiogenesis, proliferation, and anti-apoptotic pathways all of which can contribute to further hypoxia and ROS. Echinomycin inhibits the ability of HIF-1a to bind DNA. (3) Wingless-related integration site/β-catenin (Wnt/β-catenin) also drives fibroid growth and results in further hypoxia given the dysregulated proliferation. Wnt is secreted into the ECM and activates Frizzled family receptor (FZD) and co-receptor low-density lipoprotein receptor-related protein 5 (LRP5). Downstream of this receptor activation β-catenin accumulates in the cytoplasm and then translocates to the nucleus and binds T-cell factor (TCF) and lymphocyte enhancement factor (LEF) transcription factors resulting in upregulation of proteins related to proliferation, cellular differentiation, anti-apoptosis, and TGFβ activation. Methyl jasmonate (MJ) and vitamin D (VitD) both inhibit Wnt expression and signaling through FZD. Resveratrol (RSV) and niclosamide both decrease concentrations of β-catenin. (4) TGFβ inhibition presents a further pathway to target hypoxia drivers. TGFβ3 is directly upregulated under hypoxic conditions and signals through the TGFβR and subsequent activation of SMAD3. Phosphorylated SMAD3 binds phospho-SMAD2 and SMAD4 permitting translocation into the nucleus and binding to SMAD binding elements (SBEs) which increase NADPH oxidase 4 (NOX4), ECM related proteins, and cellular proliferation. NOX4 directly produces ROS superoxide and H2O2. SB431542 and vitamin D both inhibit TGFβ3 signal activity. Gonadotropin hormone-releasing hormone (GnRH) agonists down-regulate TGFβR expression. GLX351322 directly inhibits NOX4.

Currently approved therapeutic options in the United States include medical treatment for bleeding symptoms with Gonadotropin hormone-releasing hormone (GnRH) antagonists, levonorgestrel-releasing intrauterine devices, tranexamic acid, or contraceptive agents. Additionally, GnRH agonists and selective progesterone receptor modulators can be utilized for both bleeding and bulk symptom management. FDA approved procedures include uterine artery embolization, radiofrequency ablation, MRI-guided focused ultrasound, and endometrial ablation. Surgical interventions include removal of some or all fibroids via hysteroscopic or abdominal myomectomy, or removal of uterus with hysterectomy. There are currently no FDA approved medications which directly target the hypoxia pathway to treat uterine fibroids. However, as there is a growing body of evidence that hypoxia plays a significant role in the pathogenesis of leiomyomas, this represents an area for the potential development of novel therapeutics (Figure 3).

### 6.1. Antioxidant Therapies

A variety of agents targeting oxidative damage have shown benefit in pre-clinical and population-level studies of fibroid growth. Diets high in antioxidants such as, vitamin A, carotenoids, lycopene, vitamin E, and green tea extracts, are associated with decreased risk of uterine fibroid development, and there is some evidence that dietary changes can show benefits in reducing symptoms in fibroid patients [101,102]. Statins have also been shown to be effective in limiting fibroid cell proliferation, possibly via attenuation of oxidative stress and this is an area of ongoing translational and clinical research [103]. There is ongoing research into the utilization of N-acetyl cysteine in fibroid management, with clinical trials suggesting efficacy in reducing tumor volume, which is hypothesized to be secondary to effects on free radical species [104].

### 6.2. HIF-1 Inhibition

HIF-1 inhibitors have been previously developed and studied in cancers, including gynecologic malignancies. Research by Xu et al. studied in vitro and in vivo effects of HIF-1 inhibition with both echinomycin and PX-478 to reduce expression of HIF target genes. Their research demonstrated that both inhibitors were effective in attenuation of growth and induction of apoptosis of fibroids in vitro and decreased fibroid growth when assessed using in vivo mice models [105]. Echinomycin is small DNA-binding molecule in the quinoxaline antibiotic family and has been shown to specifically inhibit the activity of HIF-1 and lead to proteasomal degradation of HIF1-alpha [106–108]. Similarly, PX-478 is an orally active small molecule that has been shown to inhibit translation of HIF1-alpha in both hypoxic and normoxic conditions [109]. PX-478 is also being studied in combination with CAR-T cell therapy in cervical cancer pre-clinical models, as potent hypoxic signaling via HIF-1a can decrease CAR-T efficacy [110]. HIF-1α inhibitor KC7F2 reduced TGFβ3 expression in fibroid cell culture enforcing the cross-talk between these pathways [92]. In other organ systems multiple clinical trials are underway attempting to target HIF signaling, and the development of novel agents with specificity for organ systems is an area of ongoing research [111].

### 6.3. WNT/ β-catenin Inhibition

Simvastatin has been shown to inhibit Wnt signaling in vitro through decreasing Wnt4 expression and expression of co-receptor LRP5. An ongoing double-blind, phase 2, randomized control trial (NCT03400826) is assessing the effect of daily treatment with 3 months of simvastatin prior to hysterectomy/myomectomy for women with fibroids. Post-operative assessment of tissues has shown a significant reduction in the active form of β-catenin confirming the translational possibility of statin treatment for fibroids [112].

Vitamin D3 has also been studied in many oncologic contexts [113] and has been shown to reduce levels of Wnt4 and β-catenin, decrease expression/activation of mTOR [114], and inhibit TGFβ3 mediated fibrosis [95] in in vitro studies of leiomyoma cells. Multiple population-based association studies have found lower levels of vitamin D to be associated with higher fibroid burdens [115–117]. Similarly, methyl jasmonate, a natural cyclopentanone lipid phytohormone and antioxidant, and Resveratrol, a natural polyphenolic phytoalexin, decreased leiomyoma cell growth through inhibiting activation of Wnt/β-catenin signaling [118,119]. Other targets of Wnt signaling, inhibitor of β-catenin and TCF4 (ICAT), niclosamide, and XAV939, have also shown significant anti-proliferative effects on primary cultures of human leiomyoma cells in vitro [120]. Multiple phase 1 and 2 studies of Wnt inhibitors are being conducted in a variety of tumor types but not have yet been approved for study in leiomyoma [121].

### 6.4. TGFβ Inhibition

GnRH agonists, one of the FDA approved treatments for fibroids, have antiangiogenic properties and down-regulate TGFβR expression and downstream effects [122]; however, length of use is limited due to safety [123]. Additional studies of inhibition at various points in the TGFβ/Smad pathway have shown decreased fibroid proliferation in vitro via suspected end target inhibition of ROS generation by NOX4. Specific inhibitory agents showing efficacy included: TGFβ/Smad inhibitor SB431542, NOX4 specific inhibitor GLX351322, and HIF-1α inhibitor KC7F2 [24,92,124]. TGFβ inhibition has also been shown to decrease normal myometrial growth, though to a lesser degree than leiomyoma growth, which may be a barrier to clinical use of these inhibitors [125].

## 7. Future Directions & Conclusions

Fibroid research continues to provide key insights into hypoxia induced cell transformation, extracellular matrix pathophysiology, hypoxic cell signaling cascades, and uterine biology. Oxidative stress and subsequent hypoxia have emerged as a driver of fibroid tumorigenesis through (1) promoting myometrial stem cell proliferation, (2) causing DNA damage propelling transformation of stem cells to tumor initiating cells, (3) driving excess ECM production. Fibroid growth, persistence, and spread is propagated through hypoxia triggered cell signaling via various pathways including HIF-1, TGFβ, Wnt/β-catenin. Hypoxia persists due to fibroid loss of antioxidant function, ECM accumulation, and growth beyond adequate vascular supplies. Current clinically available treatments for fibroids do not take advantage of therapies targeting hypoxia. Growing pre-clinical and clinical studies identify ROS inhibitors, anti-HIF-1 agents, Wnt/β-catenin inhibition, and TGFβ cascade inhibitors as agents that reduce fibroid development and growth through targeting hypoxia.

The National Institutes of Health allocated $15 million for fibroid related research in 2022, putting it in the bottom 9th of 315 diseases receiving funding, and allocating a mere $0.50 - $1.25 per woman affected in the United States [126,127]. Notably, prevalence of fibroids are 2-3x greater in black and brown women compared to white women [128]. As a matter of health justice, further research and attention needs to be given to widening our understanding of treatment modalities for fibroids. Hypoxia related research and therapeutic targets are one area of interest to continue exploring in fibroids.
